# A Rotating Machinery Fault Diagnosis Method Based on Dynamic Graph Convolution Network and Hard Threshold Denoising

**DOI:** 10.3390/s24154887

**Published:** 2024-07-27

**Authors:** Qiting Zhou, Longxian Xue, Jie He, Sixiang Jia, Yongbo Li

**Affiliations:** 1 Chengdu Aircraft Design & Research Institute, Chengdu 610091, China; qitingzhou@mail.nwpu.edu.cn (Q.Z.); fishjay007@163.com (J.H.); 2 School of Aeronautics, Northwestern Polytechnical University, Xi’an 710072, China; sixiang_j@163.com (S.J.); yongbo@nwpu.edu.cn (Y.L.)

**Keywords:** gearbox, fault diagnosis, data fusion, graph convolution network, denoising

## Abstract

With the development of precision sensing instruments and data storage devices, the fusion of multi-sensor data in gearbox fault diagnosis has attracted much attention. However, existing methods have difficulty in capturing the local temporal dependencies of multi-sensor monitoring information, and the inescapable noise severely decreases the accuracy of multi-sensor information fusion diagnosis. To address these issues, this paper proposes a fault diagnosis method based on dynamic graph convolutional neural networks and hard threshold denoising. Firstly, considering that the relationships between monitoring data from different sensors change over time, a dynamic graph structure is adopted to model the temporal dependencies of multi-sensor data, and, further, a graph convolutional neural network is constructed to achieve the interaction and feature extraction of temporal information from multi-sensor data. Secondly, to avoid the influence of noise in practical engineering, a hard threshold denoising strategy is designed, and a learnable hard threshold denoising layer is embedded into the graph neural network. Experimental fault datasets from two typical gearbox fault test benches under environmental noise are used to verify the effectiveness of the proposed method in gearbox fault diagnosis. The experimental results show that the proposed DDGCN method achieves an average diagnostic accuracy of up to 99.7% under different levels of environmental noise, demonstrating good noise resistance.

## 1. Introduction

Deep learning has provided robust support for gearbox health monitoring in recent years. Since the ability of single-sensor or channel-monitoring data to effectively characterize fault characteristics is limited, it is more reasonable to employ multi-sensor collaborative monitoring [1,2,3]. The integration of data from multiple sensors in gearbox fault diagnosis offers a comprehensive, accurate, and robust approach to assessing the health and performance of gearboxes. It enhances diagnostic capabilities, enables proactive maintenance strategies, and ultimately contributes to improving the reliability and efficiency of machinery in various industrial applications.

The multi-sensor data can comprehensively reflect equipment health status through vibration, deformation, noise, etc. [4,5]. To efficiently process large volumes of data and extract deep complementary fault characteristic information, multi-sensor information fusion technology has become a key research area. Guan et al. [6] proposed a novel approach for bearing fault diagnosis, integrating multi-sensor data across various scales. They introduced a correlation kurtosis weighted fusion rule to handle vibration signals from different directions, effectively mitigating noise interference. In contrast, Sun et al. [7] devised an attention-enhanced complementary feature fusion technique tailored for heterogeneous data sources. Their method leveraged spectral Markov conversion fields to encode acoustic data and seamlessly fuse it with infrared thermal images at the data level. Additionally, Wang [8] and Yan [9], among others, delved into data-level fusion strategies. However, dealing with multi-dimensional raw data poses challenges due to the potential presence of redundant or irrelevant information, leading to computational inefficiencies. Furthermore, the temporal dependencies inherent in monitoring data from diverse sensors often contain valuable diagnostic insights that are frequently overlooked. Graph neural networks (GNNs) have emerged as a promising paradigm for processing structured data, particularly graphs. In the realm of gearbox fault diagnosis, where complex interdependencies among multi-sensor data must be addressed, GNNs offer distinct advantages. By treating each sensor reading as a node and the relationships between sensors as edges in a graph representation, GNNs can adeptly capture both temporal and spatial dependencies. This facilitates the extraction of meaningful features crucial for accurate fault detection and diagnosis. Zhao et al. [10] proposed model-assisted multi-source fusion hypergraph convolutional neural networks for intelligent few-shot fault diagnosis for the electro-hydrostatic actuator. This approach captures more multi-sensing-related information through hypergraphs. Sun et al. [11] proposed multi-sensor graph adaptive federated generalization (MGAFG) for helicopter transmission system fault diagnosis, which introduces self-supervised learning to learn the latent distribution features of target client data.

Although the methods mentioned above have made progress in the research of multi-sensor information fusion for fault diagnosis, they all operate on static graph structures. This approach may not fully capture the local dependencies and correlations within the monitoring data. Static graph neural networks are limited in their ability to adapt to changes in the data over time or capture evolving relationships between nodes in a graph. These networks typically operate on fixed graph structures, where the connections between nodes remain unchanged throughout the training and inference process [12,13]. However, static graph neural networks cannot effectively model temporal dependencies or changes in data over time. In applications where the data evolve dynamically, such as time-series data or sequential processes like natural language processing, this limitation hinders the network’s ability to capture meaningful patterns and make accurate predictions. Many real-world datasets exhibit dynamic or evolving graph structures, where nodes and edges are added, removed, or modified over time. Static graph neural networks struggle to adapt to such changes, as they require predefined graph structures during both training and inference. In addition, static graph neural networks may struggle to generalize well to unseen data or scenarios, particularly if the underlying relationships in the data change over time. This can lead to poor performance when deployed in dynamic environments or when faced with data distributions different from those seen during training. In applications with large and complex graphs, the computational overhead of retraining static graph neural networks every time the graph structure changes can be prohibitive. This limitation makes it challenging to deploy these networks in real-time or resource-constrained environments. Therefore, while static graph neural networks have shown effectiveness in modeling fixed graph structures, their inability to capture temporal dynamics and adapt to evolving data poses significant challenges in many real-world applications. As a result, there is growing interest in developing more flexible and dynamic graph neural network architectures to overcome these limitations.

In the meantime, noise is a commonly encountered factor that influences the process. Gearboxes generate mechanical vibration noise during operation, which may result from factors such as gear meshing and bearing rotation, causing interference with the data collected by sensors. Electromagnetic interference is another prevalent factor, especially in industrial environments where many electrical devices and electromagnetic signals exist, potentially disrupting the collection and transmission of sensor signals. Additionally, sensor failures themselves can lead to inaccurate or abnormal data, such as sensor malfunctions or calibration errors, thereby affecting the accuracy of fault diagnosis. Noise can lead to a decrease in the quality of sensor data, affecting their accuracy and reliability, thereby reducing the ability of graph neural networks to process and analyze the data. It can also result in the distortion or confusion of information within the sensor data, causing valid information to be mixed with noise. This can lead to misinterpretations or erroneous inferences by graph neural networks when integrating data from multiple sensors [14]. More importantly, noise may disrupt the correlations and dependencies between sensor data, making it difficult for graph neural networks to accurately capture the true relationships between data points. This can result in the inaccurate feature extraction and inference by graph neural networks when integrating data from multiple sensors, affecting the accuracy and robustness of fault diagnosis. Therefore, when conducting data fusion and model training, it is necessary to adopt appropriate noise reduction strategies to enhance the model’s resilience to noise interference, thereby improving the accuracy and reliability of fault diagnosis.

To address the aforementioned challenges, a dynamic denoising graph neural network (DDGCN) is proposed for gearbox fault diagnosis. A dynamic graph typically refers to the scenario where the nodes and edges in a graph structure change over time or based on certain conditions. Dynamic graphs can capture temporal information and evolving processes within data, allowing for a more accurate modeling and understanding of relationships between data points. In dynamic graphs, the addition, deletion, or modification of nodes and edges can reflect the evolution of data, enabling models to adapt to changes in data and adjust flexibly. In the field of gearbox fault diagnosis, the operating status of gearboxes changes over time, and there are temporal dependencies between the monitoring data from different sensors. Furthermore, dynamic graphs can capture these temporal dependencies, allowing models to consider changes in data over time, thereby better understanding and predicting the gearbox’s operating status. The relationships between sensors in gearboxes may change with operational conditions and fault situations. Dynamic graphs can capture the dynamics of these relationships, allowing models to flexibly adjust the connections and weights between sensors to better reflect the correlations between data [15,16,17,18]. The main contributions of the paper can be summarized as follows:

(1) A dynamic denoising graph neural network (DDGCN) is proposed for gearbox fault diagnosis. Dynamic graphs are constructed and applied to capture the temporal dependencies, allowing the DDGCN to consider changes in data over time and predicting the gearbox’s operating status.
(2) A learnable hard threshold denoising layer is proposed and embedded within the dynamic graph neural network. This allows the proposed method to adaptively learn thresholds to filter out noise from dynamic graph structured data, thereby improving the reliability of the graph-structured data and subsequently enhancing the accuracy of fault diagnosis.

The remainder of this work starts with the theoretical background in Section 2. The proposed DDGCN method is presented in Section 3. In Section 4, the proposed model is verified by multi-sensor multi-condition fault diagnosis experiments for the bearing and gearbox. Finally, some conclusions are given in Section 5.

## 2. Preliminaries

Graph convolution operations can be categorized into spectral domain graph convolution and spatial domain graph convolution. Spectral domain graph convolution, rooted in the principles of graph signal processing [19], initially defined graph convolution in the Fourier domain by Bruna et al. [20], leveraging the graph Laplace decomposition. Defferrard et al. [21] further advanced this concept by introducing Chebyshev polynomials to approximate spectral filters, thereby simplifying the feature decomposition and reducing the computational complexity.

The general mathematical expression of graph data is $G\left(V,E,A\right)$, where $V=\left\{v_{1},v_{2},\cdots ,v_{n}\right\}$ denotes the set of graph nodes, the number of nodes are $\left|V\right|=n$, $E=\left\{\varepsilon_{1},\varepsilon_{2},\cdots ,\varepsilon_{m}\right\}$ denotes the set of edges, and $A\in {\mathbb{R}}^{n\times n}$ denotes the graph adjacency matrix, which is a symmetric matrix. If an edge exists between graph node $v_{i}$ and node $v_{j}$, $a_{ij}\in \left(0,1\right]$ can be used to represent the edge weight, and, if there is no edge, then $a_{ij}=0$. A raw signal x defined on the graph nodes may be regarded as a vector $x\to {\mathbb{R}}^{n}$, in which $x_{i}$ is the signal value at node $v_{i}$. The degree matrix $D\in {\mathbb{R}}^{n\times n}$ of the graph diagonal represents the number of connecting edges between a node and other nodes, where the degree of node $v_{i}$ is $d_{ii}=\sum_{j=1}^{n}a_{ij}$. The graph Laplace operator for defining the spectral graph analysis is:

(1) L=D−A

Since L is a real symmetric semi-positive definite matrix, the Laplacian is indeed diagonalized by the Fourier basis, and the obtained Laplacian matrix $L_{sym}$ after spectral decomposition is:

(2) $$L_{sym}=D^{-\frac{1}{2}}LD^{\frac{1}{2}}=I_{n}-D^{-\frac{1}{2}}AD^{\frac{1}{2}}=U^{-\frac{1}{2}}\Lambda U^{\frac{1}{2}}$$

where $I_{n}$ is the identity matrix; $U=\left[u_{0},u_{1},\cdots ,u_{n-1}\right]\in {\mathbb{R}}^{n\times n}$ is the matrix consisting of the eigenvectors of L, i.e., the Fourier basis; and $\Lambda =\mathrm{diag}\left[\lambda_{0},\lambda_{1},\cdots ,\lambda_{n-1}\right]$ is the eigenvalue matrix.

The performance of node classification in the GCN heavily relies on the initial input graph structure. Typically, graph construction is based on prior knowledge or employs strategies like the kNN approach. To ensure network efficiency, we adopt the kNN algorithm to partition multi-sensor data into graph nodes using time-sliding windows. Euclidean distances are then utilized to represent the distances between node features, facilitating the establishment of internal correlations among the original multi-sensor data.

To perform node embedding on G, we define the spectral convolution operation on the graph as the inner product of the signal x and the filter $g_{\theta }$:

(3) $$g_{\theta }*x=g_{\theta }\left(U\;\Lambda \;U^{T}\right)x=Ug_{\theta }\left(\Lambda \right)U^{T}x$$

(4) $$g_{\theta }\left(\Lambda \right)=\mathrm{diag}\left(\theta \right)$$

where $\theta \in {\mathbb{R}}^{n}$ is the Fourier coefficient vector; and $g_{\theta }$ is the filter of the graph network that is the function of the eigenvalue $\left(\Lambda \right)$ of L with respect to the parameter θ.

To speed up the network training, the filter $g_{\theta }\approx \sum_{k=0}^{K-1}\theta_{k}T_{k}\left(x\right)\tilde{\Lambda }$ fitted to a K-order Chebyshev polynomial $T_{k}\left(x\right)$ is further simplified by assuming that $K=1,\;\lambda_{\max}=2$. The first-order Chebyshev approximation is performed to obtain Equation (5), and the forward propagation rule for the GCN is obtained as Equation (6) [22]:

(5) $$g_{\theta }*x\approx \theta \left({\tilde{D}}^{-\frac{1}{2}}\tilde{A}{\tilde{D}}^{\frac{1}{2}}\right)x$$

(6) $$H^{\left(l+1\right)}={\tilde{D}}^{-\frac{1}{2}}\tilde{A}{\tilde{D}}^{\frac{1}{2}}H^{\left(l\right)}\Theta^{\left(l\right)}$$

where $H^{\left(l+1\right)}$ and $H^{\left(l\right)}$ denote the graph signals of l+1 and l layers, respectively; $\tilde{A}=A+I_{n}$; $\tilde{D_{ij}}=\sum_{j}\tilde{A_{ij}}$; Θ is the convolution kernel and the value is continuously updated; $\tilde{\Lambda }=\frac{2\Lambda }{\lambda_{\max}}-I_{n}$, where $\lambda_{\max}$ is the largest eigenvalue of L; and $T_{k}\left(x\right)=2xT_{k-1}\left(x\right)-T_{k-2}\left(x\right)$ is the Chebyshev polynomial recursion, where $T_{0}\left(x\right)=0$ and $T_{1}\left(x\right)=x$.

## 3. Proposed Approach

**Figure 1:**
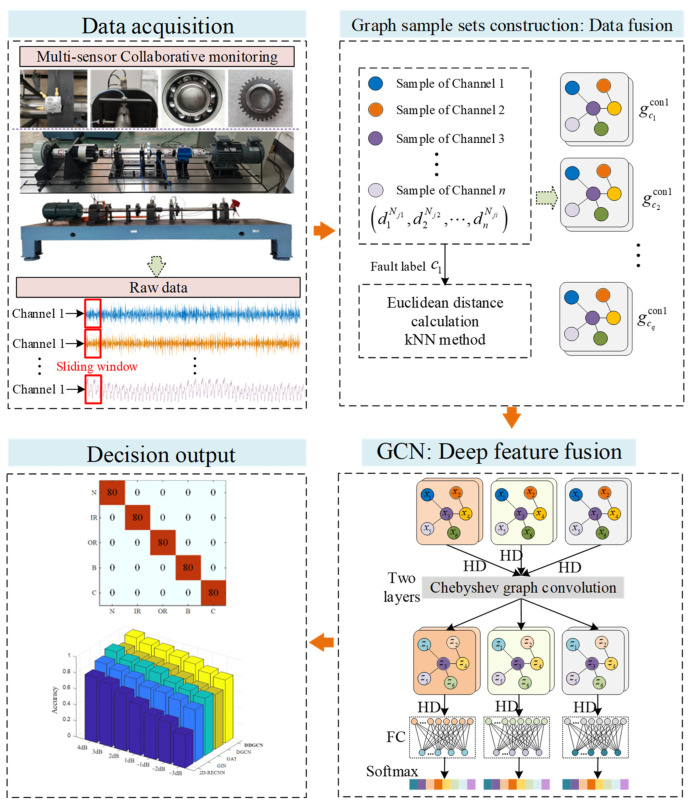
Flowchart of the proposed DDGCN method.

The proposed dual-fusion dynamic graph convolutional network (DDGCN) architecture, depicted in Figure 1, employs a data-fusion strategy to enhance fault classification reliability and performance by simultaneously integrating multi-sensor data. The DDGCN comprises two key components: the multi-sensor data fusion module and the feature fusion module. The multi-sensor data fusion module is designed to extract deep features with graph structure data fusion. The proposed method differs from the original GCN in that it can be validated on dynamic graph structures, which helps it to obtain a more robust graph-level fault characterization. The feature fusion module can learn fault representation and output the classification results. A further elaboration on these components will be provided in the subsequent sections.

### 3.1. Dynamic GCN Network

**Figure 2:**
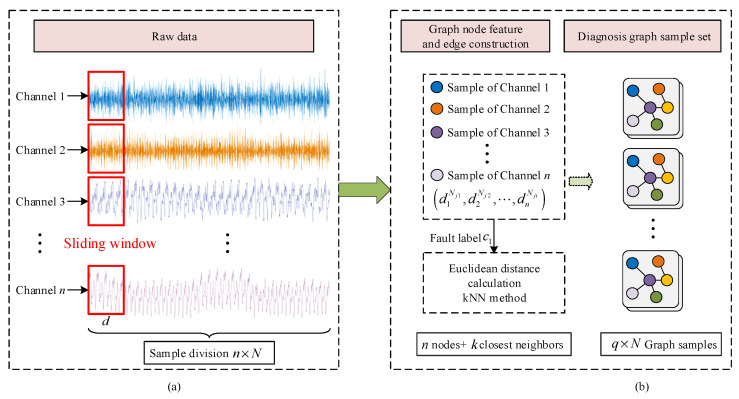
Construction of multi-sensor graph sample set. (**a**) Raw data processing, (**b**) Construction of graph sample.

The graph data constructed from the multi-sensor monitoring data can comprehensively represent the intra-data topological relationships and complementarity. As shown in Figure 2a, without any preprocessing on the raw monitoring data, N samples of length d are intercepted directly for each sensor channel using the time-sliding window, as shown in Equation (11). In the graph sample construction stage, the number of graph nodes is consistent with the number of channels. A sample of each channel is randomly selected as the node feature of the graph sample denoted as $\left(d_{1}^{N_{1}},d_{2}^{N_{2}},\cdots ,d_{n}^{N_{i}}\right)$. After label assignment, the graph samples for a specific fault type can be represented as $g_{c_{i}}^{N}$.

(7) $$N=floor(\frac{x_{i}^{\mathrm{con}j}}{d}),i\in \left[1,n\right],\mathrm{con}j\in \left[1,m\right]$$

(8) $$g_{c_{i}}^{N_{j}}=\left[\left(d_{1}^{N_{j1}},d_{2}^{N_{j2}},\cdots ,d_{n}^{N_{jn}}\right),c_{i}\right],c_{i}\in Q,j\in \left[1,N\right]$$

where n is the number of graph nodes, m denotes the number of conditions to be fused, $x_{i}$ is the monitoring data of channel i, $N_{i}$ represents the index of samples drawn from the i-th channel, and $g_{c_{i}}$ denotes the subset of graph samples labeled $c_{i}$.

Further, the kNN algorithm is employed to establish the intrinsic connections among different nodes. As shown in Equations (9) and (10), based on the Euclidean distance $D\left(d_{i},d_{j}\right)$, the k nodes closest to the central node $d_{i}$ are selected as neighboring nodes $Neigh(d_{i})$ to establish the edge relationships. As shown in Figure 2b, this process is repeated to construct graph samples for multi-sensor data in each condition, resulting in the multi-sensor graph dataset.

(9) $$Neigh(d_{i})=\mathrm{kNN}(k,d_{i},g_{c_{i}})$$

(10) $$D\left(d_{i},d_{j}\right)=\sqrt{\left(\sum_{\mu =1}^{d}{\left|d_{i}^{\mu }-d_{j}^{\mu }\right|}^{2}\right)}$$

It is worth noting that this graph construction method effectively captures the local temporal dependencies among multiple sensors, despite the possibility that the selected nodes in each graph may not precisely align across the overall time series of different sensors. Essentially, this approach allows for personalized graph structures for each graph sample, facilitating the comprehensive exploration of rich correlations among the graph nodes’ features. Moreover, compared to constructing static graph structures using the entire time series, the proposed method dynamically captures the evolving correlations among vibration time series over time. This includes variations induced by the progression of faults and subtle changes in graph structure resulting from shifts in operating conditions.

### 3.2. Hard Threshold Denoising

In this paper, we suggest incorporating the thresholding process into our architecture to learn optimal thresholding parameters, eliminating the need for treating it as a distinct step. We introduce a trainable hard-thresholding activation function, formed by combining two opposing sigmoid functions:

(11) $$HT(x)=x\left[\frac{1}{1+\exp\left(\alpha \cdot (x+b_{-})\right)}+\frac{1}{1+\exp\left(-\alpha \cdot (x-b_{+})\right)}\right]$$

where *α* is a “sharpness” factor arbitrarily fixed to 10 in this paper, and *b*_+_ and *b*_−_ are the positive and negative learnable biases acting as the thresholds on both sides of the origin. To replicate the original network without denoising, one can fix, in this layer, *b*_+_ and *b*_−_ to zero, enforcing, thereby, a linear activation. In this way, each layer has just two more parameter values that need to be learned for noise reduction. By setting *b*_+_ and *b*_−_ as learnable parameters, the proposed method can learn suitable thresholds by backward gradient propagation in an end-to-end diagnostic framework, thus obtaining a noise reduction capability at a very small parameter cost. Compared to the original GCN, the proposed method has only two more parameters to obtain additional noise reduction performance, which is a very small parameter cost compared to the huge deep-learning model. Therefore, it can be approximated that the proposed method has the same computational complexity as the GCN.

### 3.3. Overall Fault Diagnosis Framework

Subsequently, by constructing a two-layer GCN, the main network architecture is designed. Notably, the message-passing mechanism could achieve feature fusion for multi-sensor graph data. As shown in Figure 1, the model consists of two graph convolution (Gconv) layers by using the Chebyshev convolution (ChebConv), each ChebConv layer followed by a normalized layer (BN), a hard threshold denoising layer (HD), and an EdgePooling layer (EdgePooling). After the two Gconv layers, a global mean pool layer and two fully connected (FC) layers are set, with the final classification performed by the Softmax function. The probability output is calculated as follows:

(12) $$p_{i}=\frac{\exp\left(z^{i}\right)}{\sum_{c=1}^{C}\exp\left(z^{c}\right)}$$

where $z^{i}$ is the output of the i-th element in the last fully connected layer, and $p_{i}$ is the probability that the sample belongs to the *i*-th class.

The total cross-entropy loss of the multi-branch GCN model can be expressed as:

(13) $$L_{\mathrm{total}}=-\frac{1}{N_{\mathrm{total}}}\sum_{i=1}^{N_{\mathrm{total}}}\sum_{j=1}^{C}1\left\{y_{i}=j\right\}\log p_{i,j}$$

where $N_{\mathrm{total}}$ is the number of all samples, $1\left\{\cdot \right\}$ is an indicator function in which $1\left\{true\right\}=1$ and $1\left\{false\right\}=0$, $y_{i}$ is the true label of the *i-*th sample, and $p_{i,j}$ is the final probability output value of the *i-*th sample for the *j-*th class.

The training and test phases of the proposed DDGCN fault diagnosis framework can be summarized as follows:

Throughout the training phase, the framework commences by collectively gathering datasets from the transmission system under multiple sensors. Subsequently, it applies the graph construction outlined in Section 2 to fuse data from different sensors, creating a dataset which is split into the training and testing sets. Following this, the GCN model is established to fuse deep features of graph samples to derive classification probability outputs. Lastly, the DDGCN model is trained iteratively using the cross-entropy loss until a predetermined number of iterations is reached. In the testing phase, the trained DDGCN model is applied to assess using the test dataset, yielding the fault classification results. The pseudocode of the proposed method is detailed in Algorithm 1.
**Algorithm 1:** Dynamic denoising graph convolutional neural networks.**# Training process**   **Dataset collection:** Multi-sensor dataset.   **Input:** Dynamic graphs $G\left(V,E,A\right)$.   **For** *n* in *epoch* **do:**  1. The training data are generated by randomly drawing $n_{B}$ samples from dataset $G\left(V,E,A\right)$.  2. Graph representation learning with GCN.  3. Obtaining the final classification output according to Equation (12).  4. Forward propagation according to Equation (13).  5. Back propagation based on the loss function to update the model.   
**End for**
   **Output**: Optimal parameters for diagnostic models.

## 4. Experimental Verifications on Gearbox Fault Diagnosis

### 4.1. Case Study 1: NPU Dataset

#### 4.1.1. Experimental Setup and Data Description

**Figure 3:**
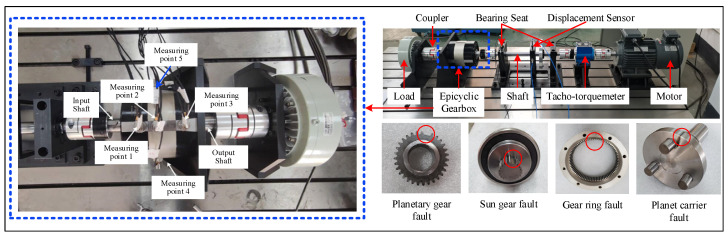
Gear transmission fault simulation experiment design. The red circles mark the locations of the damage.

Figure 3 shows the planetary gear transmission fault simulation test bench of Northwest Polytechnical University [23], which is composed of a drive motor, a load, a rotor, a bearing, a coupling, and a planetary gearbox fault kit. Five monitoring points are arranged in its shell and input/output terminals, and eleven channels of vibration signals are obtained as shown in Table 1. In this experiment, five health states of gearbox failure including NOR (Normal state), PGF (Planetary wheel failure), SGF (Sun wheel failure), GRF (Gear ring failure), and PCF (Planetary carrier failure) are designed as shown in Figure 3. The sampling frequency is 16,000 Hz, and the sampling time is 20 s. The segmentation of the data sample is consistent with the former case. Typical gear failures and operating conditions can be simulated using this test bed, allowing usable health detection signals to be obtained using high-precision sensing equipment. In signal acquisition, the deployment points of multiple sensors vary in distance from the faulty part, so their acquired monitoring signals can be viewed as physical descriptions of different perspectives of the gear’s state of health.

#### 4.1.2. Implementation Details

To verify the superiority of our DDGCN model, four comparison methods are designed: (1) 2D-RECNN—directly splicing multi-sensor data to construct a 2D dataset, and then using RESNET to perform deep feature extraction and fault diagnosis; (2) Graph Isomorphism Network (GIN) [24]—by iteratively aggregating operations on nodes and their neighboring nodes to generate node representations; (3) Graph Attention Network (GAT) [25]—using attention mechanisms to learn the relationships between nodes, dynamically modeling the weights between nodes; and (4) Dynamic GSN (DGCN)—based on the proposed DDGCN, the hard threshold denoised layers are removed.

To ensure comparability, the network structure and its basic parameter settings involved in the above methods are consistent with the proposed DDGCN. Note that the GAT and GIN methods use static graph data to perform multi-sensor data fusion and gearbox fault diagnosis. The network architectures of comparison methods share the same number of network layers with the GCN. Three fault diagnosis tasks were constructed using three different quantities of sensor monitoring data, each containing 7 (point 1, 2, and 4), 9 (point 1, 2, and 3), and 11 (all points) sensors, respectively. The total number of graph samples for the five health states is 2000, and each graph sample contains five graph nodes, with 5×1024 points as the graph node features. In the kNN algorithm, the parameter k is set to 4 to establish edge relationships. Since the structure of the graph is dynamically changing, the parameter *k* should, in principle, be chosen in such a way as to retain sufficient room for variation. Taking the size of the graph into account, the parameter k is set to 4 to establish edge relationships. The learning rate is set to 0.001, the batch size is 32, the number of training epochs is 300, and the size of Chebyshev convolution kernels is 2. At the same time, a dropout layer is added between the two layers of FC to prevent overfitting with a zeroing probability of 0.5.

#### 4.1.3. Diagnosis Result and Discussion

Each method was tested ten times in all tasks to avoid random factors. Table 2 reports the statistical results of methods utilizing multi-sensor data. It can be seen that the proposed method achieves the highest average accuracy of 99.36%, surpassing the four comparison methods by 20.60%, 9.10%, 2.13%, and 9.50%, respectively. Specifically, the 2D-RECNN method only achieved an accuracy of 78.76%, significantly lower than the other methods. This is because the method merely concatenates the monitoring data from multiple sensors and fails to establish their internal correlations. GIN, utilizing iteratively aggregating operations, obtained a more effective node representation, resulting in an accuracy of 90.26%, but still significantly lagging behind the proposed method. This is attributed to its use of static graph structures for feature extraction, which cannot capture the local temporal relationships in the monitoring data from multiple sensors. Despite iteratively aggregating node features, the disadvantage of static graph structures weakens its performance in fault representation learning. Similarly, GAT employs attention mechanisms for node feature extraction, but, under the premise of all graph samples sharing a static edge connection, the attention mechanism fails to exert its effectiveness. As a result, the performance obtained is inferior to that of the proposed method.

#### 4.1.4. Feature Visualization Analysis

**Figure 4:**
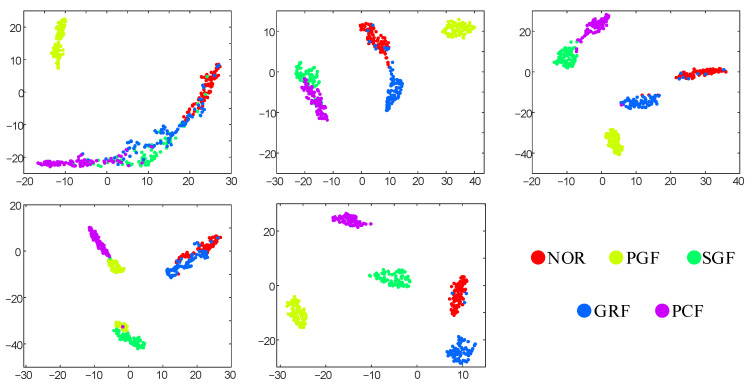
t-SNE feature visualization of all methods in 7-sensor tasks.

To intuitively demonstrate the feature extraction capabilities of all methods for multi-sensor monitoring data, we conducted t-SNE dimensionality reduction on the features obtained from the last fully connected layer in Task 1. From Figure 4, it can be observed that the 2D-RECNN method exhibits the worst dimensionality reduction visualization effect, with a significant amount of sample misclassification. In method 2, there is a certain degree of confusion between the normal samples and GRF samples, as well as between the SGF samples and PCF samples. Method 3 achieves relatively good classification results, but its inter-class distances are small, indicating that this method cannot capture fault features with strong class discrimination. DGCN, lacking a denoising layer, easily obtains features that are highly sensitive to fault interference, resulting in a poor dimensionality reduction performance. The proposed method achieves the best dimensionality reduction effect, characterized by minimal intra-class distances and relatively large inter-class distances.

#### 4.1.5. Noise Resistance Analysis

**Figure 5:**
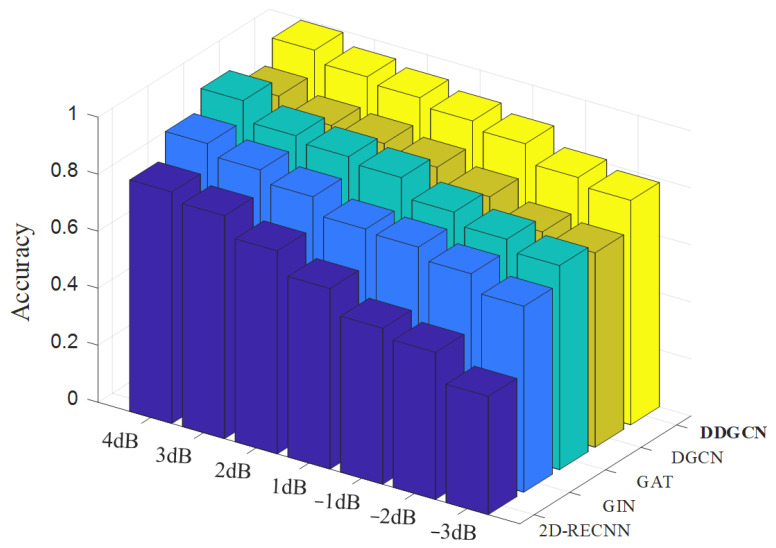
Fault recognition accuracies of compared methods at seven noise levels.

In industrial settings involving rotating machinery, noise is a pervasive challenge that is difficult to mitigate. Therefore, Gaussian white noise with different signal-to-noise ratios (SNRs) is injected into the raw monitoring data to analyze the noise resistance of the diagnosis methods. In the task of nine-sensor fusion, a total of seven noise levels with a range of −3 dB~4 dB are set, and the diagnostic results of each comparison method are reported in Table 3 and Figure 5. It can be observed that, with the increase in noise intensity, the performance of all methods declines. Among them, the performance degradation of 2D-RECNN reaches 39.7%, indicating that using graph-structured data can enhance the noise robustness of the model to a certain extent. This is because graph features depend on iterative calculations between nodes and their relationships, rather than solely on node features. It is worth mentioning that the proposed method achieves the best accuracy under all signal-to-noise ratios (SNRs). Particularly, as the noise intensity increases, the advantage of the proposed method’s noise resistance becomes increasingly evident. At an SNR of −3 dB, the proposed method’s accuracy leads the comparative methods by over 7.1%. This analysis indicates that the proposed method exhibits superior noise robustness.

### 4.2. Case Study 2: XJTU Dataset

#### 4.2.1. Experimental Setup and Data Description

**Figure 6:**
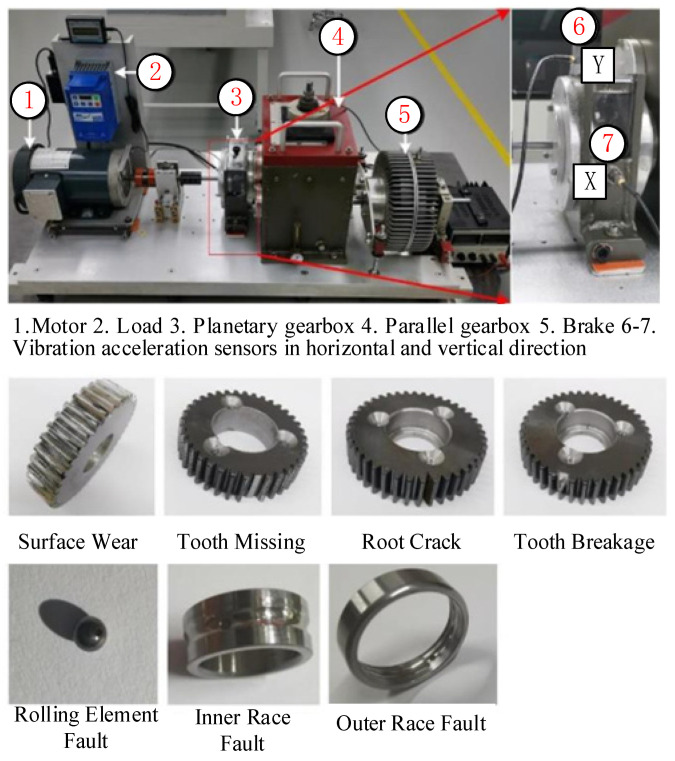
Gearbox fault test bench. X and Y indicate the positions of the two sensors.

Even when the data originate from a single sensor, the proposed method can still utilize single-channel samples for graph sample construction, thereby mining the local temporal dependencies and variation trends in individual sensor-monitoring data. Therefore, to further demonstrate the multi-channel feature extraction capability of the DDGCN, a single-channel experimental dataset was employed for validation. The experimental platform of the XJTU Gearbox dataset [26] comprises a driving motor, a load application device, a planetary gearbox, a parallel gearbox, a brake, and two sensors. Specifically, the motor is powered by a three-phase alternating current (230 V, 60/50 Hz). Two PCB352C04 vibration accelerometers are installed in both the horizontal and vertical directions of the planetary gearbox. Eight types of fault modes are pre-fabricated on the planetary gearbox, including four gear fault modes and four bearing fault modes: (1) surface wear; (2) tooth missing; (3) root crack; (4) tooth breakage; (5) rolling element fault; (6) inner race fault; (7) outer race fault; and (8) mixed inner and outer race fault. Including the normal state, there are a total of nine modes in the dataset. The motor speed is set to 1800 rpm, and the sampling frequency is set to 20,480 Hz. The construction of the gearbox test bench and the seven fault components are shown in Figure 6. In data sample division, 400 samples are divided using a sliding window with a length of 1024. Taking out one sample randomly from each channel, 400 graph samples for each health state can be constructed. Then, 80% of the graph samples are randomly selected as the training set, and 20% are selected as the test set. In order to verify the diagnostic ability of the base method in small-sample scenarios, two additional training sample ratios are designed, 60% and 40%, respectively.

#### 4.2.2. Diagnosis Result and Discussion

Each method was tested ten times in all tasks to avoid random factors. Table 4 reports the statistical results of methods utilizing multi-sensor data. The proposed method achieves the highest average accuracy of 94.6%, surpassing the four comparison methods by 27.8%, 18.6%, 10.3%, and 9.8%, respectively. Specifically, the 2D-RECNN method only achieved an accuracy of 66.8%, significantly lower than the other methods. This suggests that the diagnostic ability of non-graph structured data in small-sample scenarios is very limited. The performance of GIN and GAT still lags behind that of the proposed method, which indicates that dynamic graph data in small-sample scenarios can utilize limited data resources more efficiently, thus obtaining more meaningful frontal fault characterization and improving fault diagnosis accuracy. Although the DGCN employs a dynamic graph structure for the computation, the presence of noise may lead to unstable adjacency matrices and, thus, noisy large intraclass variance, which is not conducive to the learning of stable fault representations by the graph network, as the graph samples are constructed from a single sensor. Therefore, this method lags behind the proposed method. It can be seen that, with only 20% of the training samples, the proposed method still has a high accuracy of 90.2%, which fully reflects the powerful fault feature extraction capability of the proposed method. In summary, the proposed method still has a good fault representation extraction capability in single-sensor fusion diagnosis.

## 5. Conclusions

This article proposes a dynamic denoising graph neural network (DDGCN)-based fault diagnosis method for transmissions, which realizes the local time-series dependent acquisition of sensor monitoring data through the construction of dynamic graph structure data. The main idea of this method is to effectively fuse multiple sensors monitoring time-series data through a graph convolutional neural network. In addition, a learnable hard threshold noise reduction layer is designed to effectively improve the anti-noise performance of the network. The proposed method is validated using two transmission datasets, and the experimental results show that the proposed method achieves the highest fault diagnosis accuracy of 96.0%. The feature visualization shows that the proposed method can learn fault features with excellent discrimination. Compared with several start-of-the-art methods, the proposed DDGCN method has a better fault feature extraction capability in noisy and small-sample scenarios.

## Figures and Tables

**Table 1:** Design of multi-sensor monitoring scheme for the planetary gearbox.

Measuring Point Number	Location	Direction	Channel Number	Data Type
1	Near the input shaft	Radial	Y1	Vibration signal
		Tangential	X1	
		Axial	Z1	
2	Inner gear ring	Radial	Y2	
		Tangential	X2	
		Axial	Z2	
3	Near the output shaft	Radial	Y3	
		Tangential	X3	
		Axial	Z3	
4	Inner gear ring position, −90° distribution from measurement point 2	Axial	Y4	
5	Inner gear ring position, +90° distribution from measurement point 2	Axial	Y5	

**Table 2:** The fault recognition results of methods with multiple sensors in NPU dataset.

Tasks	2D-RECNN	GIN	GAT	DGCN	DDGCN
7 sensors	72.1%	90.1%	95.6%	85.2%	99.1%
9 sensors	81.2%	90.3%	97.5%	91.2%	99.4%
11 sensors	83.0%	92.4%	98.6%	93.2%	99.6%
Average accuracy	78.76%	90.26%	97.23%	89.86%	99.36%
Standard deviation	4.2%	3.6%	2.1%	0.3%	0.2%

**Table 3:** The fault recognition results of all methods at seven noise levels.

SNR	Method				
	2D-RECNN	GIN	GAT	DGCN	DDGCN
4 dB	81.2%	90.3%	97.5%	91.2%	99.4%
3 dB	78.2%	86.4%	90.5%	86.2%	95.4%
2 dB	71.2%	82.3%	88.5%	85.2%	93.4%
1 dB	63.3%	76.3%	86.5%	82.2%	90.6%
−1 dB	54.7%	75.3%	79.7%	77.2%	87.9%
−2 dB	51.6%	71.3%	75.5%	70.2%	81.4%
−3 dB	41.5%	65.2%	71.6%	68.2%	78.7%

**Table 4:** The fault recognition results of all methods on XJTU dataset.

Tasks	2D-RECNN	GIN	GAT	DGCN	DDGCN
80% of training samples	85.1%	95.1%	98.5%	94.0%	99.5%
60% of training samples	78.2%	86.4%	91.5%	85.2%	95.4%
40% of training samples	61.5%	70.3%	85.6%	84.2%	93.1%
20% of training samples	42.4%	53.5%	61.4%	75.6%	90.2%
Average accuracy	66.8%	76.3%	84.3%	84.8%	94.6%
Standard deviation	3.8%	2.9%	1.5%	0.9%	0.7%

## Data Availability

The original contributions presented in the study are included in the article, further inquiries can be directed to the corresponding author.
